# Are the Rates of Hypertension and Diabetes Higher in People from Lower Socioeconomic Status in Bangladesh? Results from a Nationally Representative Survey

**DOI:** 10.1371/journal.pone.0127954

**Published:** 2015-05-27

**Authors:** Md. Ismail Tareque, Atsushi Koshio, Andrew D. Tiedt, Toshihiko Hasegawa

**Affiliations:** 1 Department of Population Science and Human Resource Development, University of Rajshahi, Rajshahi, Bangladesh; 2 The Graduate School of Project Design, Tokyo, Japan; 3 United States Department of Justice, Washington, DC, United States of America; 4 Department of Health Policy and Management, Nippon Medical School, Tokyo, Japan; National Institute of Health, ITALY

## Abstract

**Objective:**

A well-established belief regarding inequalities in health around the world is that hypertension and diabetes are higher in groups of lower socioeconomic status. We examined whether rates of hypertension, diabetes, and the coexistence of hypertension and diabetes are higher in people from a lower socioeconomic status than in those from a higher socioeconomic status in Bangladesh.

**Methods:**

We investigated a nationally representative dataset from the 2011 Bangladesh Demographic and Health Survey with objective measures for hypertension and diabetes. A wealth index was constructed from data on household assets using principal components analysis. Chi-square tests and logistic regressions were performed to test the associations between wealth level, hypertension and diabetes.

**Findings:**

People from the highest wealth quintile were significantly more likely to have hypertension (Adjusted odds ratios [AOR] = 1.65, 95% confidence interval [CI] = 1.22-2.25), diabetes (AOR = 1.81, 95% CI = 1.21-2.71), and the coexistence of hypertension and diabetes (AOR = 2.17, 95% CI = 1.05-4.49) than people from the lowest wealth quintile. The odds of having hypertension, diabetes, and their coexistence were higher for older people, women, people who engaged in less physical labor, and people who were overweight and obese.

**Conclusion:**

Wealthier people, particularly people from the fourth and highest wealth quintiles, should be careful to avoid unhealthy lifestyles to prevent hypertension and diabetes. Health policy makers and planners are urged to target wealthier strata in terms of hypertension and diabetes initiatives while paying special attention to older people, women, people who engage in less physical labor, and individuals who are overweight.

## Introduction

Socio-economic disparity is a key public health concern [1]. A well-established belief regarding inequalities in health around the world, particularly in developed regions, is that mortality rates, poorer self-assessment of health, illness, disability, hypertension, diabetes, and non-communicable diseases are higher in groups of lower socioeconomic status [2–12], whether socioeconomic status is measured in terms of income, education, occupational status, or household assets. A recent study on inequality in disability in Bangladesh reported that people from poor families have a greater likelihood of reporting disabilities than people from wealthier families [13]. To our knowledge, the association between wealth level and health in terms of hypertension and diabetes has never been tested in Bangladesh.

High blood pressure acts as one of the contributing and intermediate risk factors for developing coronary heart disease, stroke, and kidney disease. The leading causes of mortality in the world as of 2004 were high blood pressure (13%), tobacco use (9%), high blood glucose (6%), physical inactivity (6%), and being overweight and obese (5%). These factors are responsible for increasing the risk of non-communicable diseases such as heart disease, diabetes and cancers [14]. A recent meta-analysis reported that out of the 1 billion people with hypertension globally, two-thirds reside in low- and middle-income countries. This study reported that higher income, household assets or social class were positively associated with hypertension in rural South Asia whereas no association was detected in rural East Asia [15]. Using data from adults aged 25 to 64 years in Matlab, Bangladesh another study reported that hypertension is more prevalent among females than in males (21% vs 13%, respectively) and increases with age [16]. A meta-analysis of studies between 1995 and 2010 on cardiovascular diseases and diabetes in Bangladesh reported that hypertension was higher among females, elderly, urban people, and working professionals than their counterparts. However, diabetes was higher in males than females, and in urban areas than rural areas, but not statistically significant [17]. On the other hand, a cross-sectional study of 402 adults aged 30 years and older in Dhaka, Bangladesh described a higher proportion of females than males with diabetes [18]. The same study also reported that older age, female sex, being overweight or obese, scoring high on a wealth index and family history of diabetes were positively associated with diabetes or metabolic syndrome. The coexistence of hypertension and diabetes was also discussed as increasing the risk of preeclampsia in pregnant women and end-organ disease among children [19]. In patients with diabetes, hypertension confers an enhanced risk of cardiovascular disease [20]. Therefore, using a nationally representative survey, we examined the critical question of whether people from lower socioeconomic status have higher likelihood of having hypertension, diabetes, and the coexistence of hypertension and diabetes than those from higher socioeconomic status in Bangladesh.

## Methods

### Data source

This study utilizes data from a nationally representative survey, the 2011 Bangladesh Demographic and Health Survey (BDHS). The 2011 BDHS is the sixth Demographic and Health Survey (DHS) undertaken in Bangladesh. The sampling design, questionnaires, and data collection procedures of the 2011 BDHS are described elsewhere in detail [21]. The 2011 BDHS is the first national survey that included biomarker measurements for blood pressure and fasting blood glucose using a household questionnaire for people aged 35 years and over.

The 2011 BDHS survey is based on a two-stage stratified sample of households. It collected a nationally representative sample covering the entire population residing in private dwelling units in Bangladesh. Based on the sampling design, a total of 17,964 households were selected, and 17,511 were found to be occupied. Interviews were successfully completed in 17,141 households, or 98% of all the occupied households. In one in three households, all men and women aged 35 and older (4524 men and 4311 women) were selected for blood pressure and blood glucose measurement. Among them, 3876 men and 3963 women participated in the blood pressure measurement, and 3721 men and 3822 women participated in the blood glucose measurement. We selected those who gave their full consent to participate in both blood pressure and blood glucose measurements and excluded pregnant cases and cases with missing observations for all the variables used in this study; consequently, the final study sample size was 7499 (3715 men and 3784 women).

The 2011 BDHS survey also provides data on Body Mass Index (BMI) for 5703 individuals. When we investigated BMI, we selected those who gave their full consent to participate in blood pressure, blood glucose, weight and height measurements and excluded cases who were pregnant and the cases with missing observations for all the variables used in this study; consequently, the study sample size became 5223 (3620 men and 1603 women).

### Measures

#### Outcomes

We examined three outcome variables in the current study, namely, hypertension, diabetes, and the coexistence of hypertension and diabetes.

The 2011 BDHS used the LIFE SOURCE UA-767 Plus blood pressure monitor model to measure blood pressure. Three measurements of both systolic blood pressure (SBP) and diastolic blood pressure (DBP) were taken during the survey at approximately 10-minute intervals. The average of the second and third measurements was used to report the respondent’s blood pressure values. The respondents were also asked whether s/he is currently taking antihypertensive medication to lower their blood pressure. We considered an individual to have hypertension when s/he had blood pressure levels ≥ 140 mmHg SBP or ≥ 90 mmHg DBP, or s/he was taking antihypertensive medication to lower their blood pressure.

Blood glucose in capillary whole blood, obtained from the middle or ring finger from subjects after an overnight fast, was measured using the HemoCue 201+ blood glucose analyzer. The HemoCue 201+ analyzer displayed the blood glucose measurements in milligrams per deciliter, which was converted to millimoles per liter [22]. The blood glucose measurements in whole blood were further adjusted by multiplying each value by 1.11 to produce the plasma glucose equivalent values [23]. Although the World Health Organization recommends that venous plasma should be used for measuring the glucose concentration in blood [24], capillary sampling (whole blood obtained from a finger prick) is widely used, particularly in resource-limited countries such as Bangladesh. We classified an individual as having diabetes if s/he had a plasma glucose value ≥ 7.0 mmol/L.

We considered an individual to have both hypertension and diabetes if s/he was found to have blood pressure levels ≥ 140 mmHg SBP or ≥ 90 mmHg DBP or s/he was currently taking antihypertensive medication to lower their blood pressure, and s/he had a plasma glucose value ≥ 7.0 mmol/L.

#### Wealth index

Using principal components analysis, the wealth index was constructed from data on household assets including ownership of durable goods (such as televisions and bicycles) and dwelling characteristics (such as source of drinking water, sanitation facilities, and construction materials), based on the assumption that the wealth of a household is related to an underlying continuum of economic status in the 2011 BDHS. The wealth index was created in three steps and takes into account differences between urban and rural areas in scores and indicators of wealth [21]. At first, categorical variables (household assets) were transformed into separate dichotomous (0 or 1) indicators. Second, separate factor scores were produced for households in urban and rural areas using area-specific indicators. The third step combined the separate area-specific factor scores to produce a nationally applicable combined wealth index by adjusting area-specific scores through a regression on the common factor scores. This three-step procedure permits greater adaptability of the wealth index in both urban and rural areas in Bangladesh. Once the index was computed, national-level wealth quintiles (from lowest to highest) were obtained by assigning the household score to each de jure household member, ranking each person in the population by his or her score, and then dividing the ranking into five equal categories, each comprising 20% of the population [21].

The wealth index is a measure that has been used in many DHS and other country-level surveys to measure inequalities in health outcomes. It serves as an indicator of wealth that is consistent with expenditure and income measures. Compared with expenditure measures, it is reported to be an easier measure of economic status to collect and produces superior, more believable results and equal or greater distinctions in health outcomes [25].

#### Covariates

Several covariates that have been theoretically and empirically linked to hypertension and diabetes [2, 3] are included in the present study: age (grouped within the following categories: 35–39, 40–44, 45–49, 50–54, 55–59, 60–69, and ≥70 years), currently married (yes or no, with no including divorced, separated, deserted, widowed, and single), schooling (grouped as none, primary incomplete, primary complete, secondary incomplete, and secondary complete or higher), less physical labor (yes or no), place of residence (rural or urban), and BMI (grouped as thin with BMI < 18.5, normal with BMI = 18.5–24.9, overweight with BMI = 25.0–29.9, and obese with BMI ≥ 30.0). Primary education indicates completion of grade 5, secondary education indicates completion of grade 10, and higher education indicates education beyond grade 10 in the 2011 BDHS [21]. The covariate of less physical labor includes the following professional and white collar occupations: doctor, lawyer, dentist, accountant, teacher, nurse, family welfare visitor, big businessperson, and imam or religious leader.

### Analysis plan

Gender differences in descriptive statistics of the study respondents were performed by Chi-square tests. Next, using Chi-square tests, we examined the differences in the proportion of hypertension, diabetes and the coexistence of hypertension and diabetes by selected socio-demographic characteristics. Then, the associations between the wealth index and hypertension, diabetes and the coexistence of hypertension and diabetes were examined with a series of logistic regression models.

Three logistic regression equations were fit for each of the outcomes. The first set of equations for the three outcomes established a bivariate association between the outcomes and wealth quintile by showing results that were unadjusted; results are presented in the form of odds of having hypertension, diabetes, and the coexistence of hypertension and diabetes, respectively. In the second set of equations for the three outcomes, an attempt was made to examine possible mechanisms driving any association by adjusting for age, gender, marital status, schooling, physical labor, and place of residence. In the third set of equations for the three outcomes, the associations were adjusted for all covariates. Furthermore, the effects of an interaction between age and gender and of an interaction between gender and less physical labor in the fully adjusted models (the third set of equations) were tested separately; no significant effect was found (results not shown). Multicollinearity in the logistic regression analyses in our study was checked by examining the standard errors for the regression coefficients. A standard error larger than 2.0 indicates numerical problems such as multicollinearity among the independent variables [26]. The net significance of the wealth quintile in all equations was estimated by calculating the difference in the log-likelihood statistic (Δ-2X LL) between equations that did and did not contain the wealth categories (the latter not shown).

Finally, the predicted probabilities of having hypertension, diabetes, and the coexistence of hypertension and diabetes were estimated across wealth levels and presented in graphs. To determine the probability, the values of all variables for the fully adjusted models, except for wealth categories, were held constant, and the mean sample probability of having hypertension, diabetes, and the coexistence of hypertension and diabetes were calculated. As such, the result can be interpreted as the probability that an otherwise average respondent would have hypertension, diabetes, and the coexistence of hypertension and diabetes. The entire analysis of the study was performed with Stata/SE version 12.1 (StataCorp LP, College Station, Texas, USA), and a sample weight was applied to accommodate the survey design. In particular, the ‘svy’ suite of commands was used to specify the cluster sampling design, sampling weights, stratification, and the calculation of standard errors. These commands used the Taylor series linearization method to estimate confidence intervals around prevalence estimates.

### Ethical considerations

Data collection procedures for the 2011 BDHS were approved by the ICF International Institutional Review Board. Informed consent was obtained from all respondents in the survey before asking questions, and separately before obtaining measurements of hypertension, diabetes, weight and height [21]. Respondents who did not provide consent were excluded from the analysis for the current study.

## Results

Table 1 shows descriptive information about the study respondents by sex. A higher percentage of men than women were found to be currently married. Further, a higher percentage of men than women had a secondary or higher education and were engaged in professional occupations requiring less physical labor; doctors, lawyers, dentists, accountants, teachers, nurses, family welfare visitors, big businessperson, and imams or religious leaders. A higher percent of women than men were, however, found to be overweight and obese; and more women (32%) than men (20%) had hypertension. There was no statistically significant difference between genders in having diabetes (10% for both sexes). However, a statistically significant difference between genders was found for individuals having both hypertension and diabetes (3% for men and 4% for women).

**Table 1:** Sample characteristics^a^.

Characteristics	Men (N = 3715)	Women (N = 3784)
	%	%
**Age group**		
35–39	16.84	20.43
40–44	16.36	18.65
45–49	15.10	15.78
50–54	15.96	11.03
55–59	8.01	9.77
60–69	14.87	13.16
70+	12.87	11.18
**Currently married** ^**b**^		
No	2.99	28.32
Yes	97.01	71.68
**Schooling**		
None	36.45	58.29
Primary incomplete	25.07	20.46
Primary complete	11.94	7.92
Secondary incomplete	14.86	8.66
Secondary complete or higher	11.68	4.68
**Less physical labor**		
No	90.65	99.70
Yes	9.35	0.30
**Place of residence**		
Rural	76.19	77.09
Urban	23.81	22.91
**Body Mass Index (BMI)** ^**c**^		
Thin (BMI < 18.5)	29.02	36.31
Normal (BMI = 18.5–24.9)	61.87	50.30
Overweight (BMI = 25.0–29.9)	8.42	10.62
Obese (BMI ≥ 30.0)	0.69	2.78
**Having hypertension**		
No	80.47	67.94
Yes	19.53	32.06
**Having diabetes**		
No	90.40	90.50
Yes	9.60	9.50
**Having both hypertension and diabetes**		
No	97.36	95.79
Yes	2.64	4.21

^a^ Gender differences in all characteristics except Place of residence and Having diabetes are significant at p < 0.01.

^b^ A total of 103 men and 1077 women were divorced, separated, deserted, or widowed, and 14 men and 12 women were single.

^c^ Total number of observations were 5223 (3620 men and 1603 women).

Table 2 shows the percentage of respondents with hypertension and diabetes by separate socio-demographic categories. Significant differences were found between wealth quintiles; although similar percentages of people in the lowest through middle wealth quintiles had hypertension, diabetes, and both hypertension and diabetes, respectively, a significantly higher percentage of people from the fourth and highest quintiles had hypertension, diabetes, and both hypertension and diabetes. There was a significant, positive association between age and having hypertension, having diabetes, or their coexistence. Older people had a higher percentage of hypertension, diabetes, and both hypertension and diabetes than younger people with the exception of the age group 55–59 years for diabetes and the coexistence of hypertension and diabetes. Currently married individuals exhibited lower percentages for all the outcomes than those who were not currently married. People with some schooling had a significantly higher percentage of hypertension, diabetes, or the coexistence of hypertension and diabetes than people with no schooling, except for the hypertension among the primary incomplete groups. Those who engaged in less physical labor had a significantly higher percentage of all the outcomes than their counterparts. People from urban areas had higher percentages of all the outcomes of hypertension, diabetes, and the coexistence of hypertension than people from rural areas. Finally, people who were overweight and obese had a significantly higher percentage of all the outcomes than their counterparts.

**Table 2:** Percent of individuals having hypertension, diabetes and the coexistence of hypertension and diabetes by socio-demographic characteristics (N = 7499).

	Hypertension		Diabetes		Both hypertension and diabetes	
	%	95% CI	%	95% CI	%	95% CI
**Wealth quintile**						
Lowest	18.94	16.62–21.50	6.82	5.34–8.67	1.50	0.92–2.45
Second	21.90	19.55–24.46	6.67	5.21–8.50	1.74	1.08–2.79
Middle	22.46	20.00–25.13	6.90	5.57–8.50	1.92	1.28–2.88
Fourth	27.96	25.52–30.53	9.63	8.00–11.54	3.54	2.62–4.77
Highest	37.06	34.41–39.78	17.17	15.05–19.52	8.12	6.67–9.85
p value	<0.001		<0.001		<0.001	
**Age group**						
35–39	14.61	12.64–16.83	7.67	6.10–9.59	1.75	1.11–2.76
40–44	19.94	17.57–22.55	8.41	6.84–10.31	1.95	1.27–3.00
45–49	24.47	21.81–27.34	9.85	8.05–11.99	3.78	2.76–5.15
50–54	26.37	23.38–29.59	9.29	7.49–11.47	3.16	2.15–4.62
55–59	29.87	26.21–33.82	15.48	12.69–18.75	6.99	5.11–9.50
60–69	35.17	31.90–38.59	9.47	7.53–11.85	4.55	3.33–6.20
70+	39.28	35.72–42.96	9.73	7.71–12.20	4.15	2.94–5.82
p value	<0.001		<0.001		<0.001	
**Currently married**						
No	41.16	38.16–44.23	10.14	8.36–12.25	5.43	4.09–7.18
Yes	22.99	21.69–24.33	9.44	8.59–10.36	3.06	2.62–3.58
p value	<0.001		0.480		<0.001	
**Schooling**						
None	26.86	25.06–28.73	7.58	6.55–8.76	2.55	1.99–3.26
Primary incomplete	21.64	19.46–23.99	9.71	8.20–11.47	3.04	2.28–4.04
Primary complete	28.30	24.69–32.22	11.31	9.08–14.01	3.96	2.73–5.72
Secondary incomplete	27.05	23.74–30.64	13.26	10.98–15.92	5.88	4.44–7.75
Secondary complete or higher	27.04	23.36–31.07	13.08	10.41–16.30	5.56	3.66–8.35
p value	0.003		<0.001		<0.001	
**Less physical labor**						
No	25.61	24.28–26.98	9.16	8.34–10.05	3.23	2.77–3.77
Yes	30.74	26.10–35.79	17.31	13.65–21.72	7.50	5.12–10.85
p value	0.042		<0.001		<0.001	
**Place of residence**						
Rural	23.76	22.30–25.29	8.42	7.53–9.41	2.68	2.21–3.24
Urban	32.71	30.31–35.20	13.23	11.46–15.24	5.93	4.87–7.20
p value	<0.001		<0.001		<0.001	
**Body Mass Index (BMI)** ^**a**^						
Thin (BMI < 18.5)	18.46	16.34–20.79	6.33	4.98–8.01	0.92	0.50–1.70
Normal (BMI = 18.5–24.9)	26.17	24.41–28.00	9.93	8.75–11.24	3.73	3.02–4.59
Overweight (BMI = 25.0–29.9)	43.76	39.12–48.52	19.47	15.56–24.08	10.30	7.72–13.61
Obese (BMI ≥ 30.0)	66.97	54.19–77.65	18.92	11.22–30.12	12.50	6.39–23.00
p value	<0.001		<0.001		<0.001	

Abbreviations: CI, confidence interval.

p values are from Chi-square tests.

^a^ Total number of observations were 5223 (3620 men and 1603 women).

**Fig 1:**
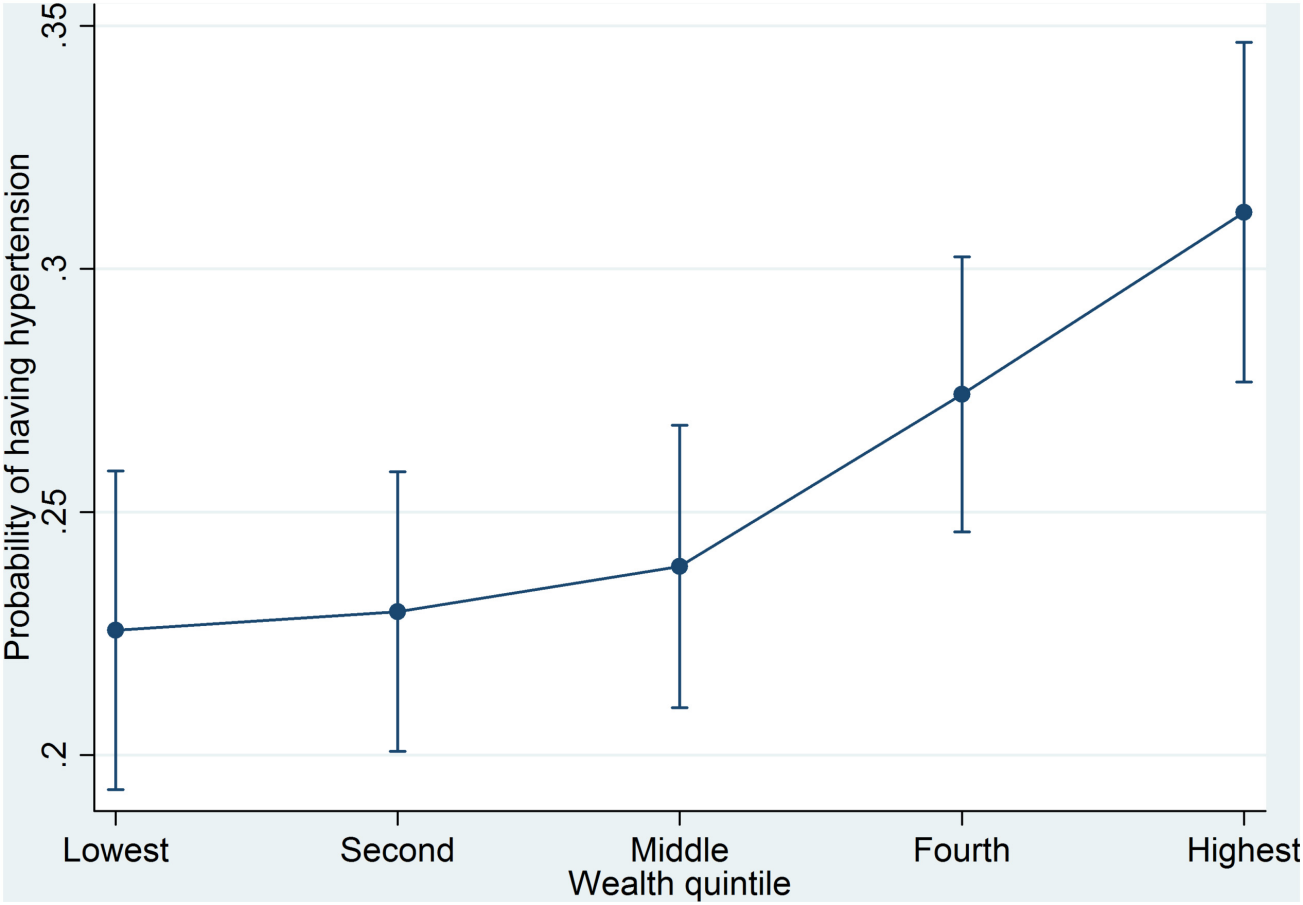
Predicted probabilities of having hypertension by wealth quintile.

Consistent with the tests of association (Table 2), the logistic regression (Table 3), revealed a positive association between wealth and hypertension with the odds increasing from the lowest to highest wealth quintiles. Individuals from the fourth and highest wealth quintile were found to be more likely to have hypertension than the individuals from the lowest wealth quintile across the boards. These results are demonstrated by the predicted probabilities from the fully adjusted model in Fig 1. In the fully adjusted model for hypertension, people from the fourth wealth quintile were significantly 1.34 times (95% CI = 1.03–1.75) more likely to have hypertension than people from the lowest wealth quintile; and people from the highest wealth quintile were 1.65 times (95% CI = 1.22–2.25) more likely to have hypertension than people from the lowest wealth quintile.

**Table 3:** Odds ratios of having versus not having morbidity in Bangladesh (N = 7499)^a^.

	Hypertension		Hypertension		Hypertension^d^		Diabetes		Diabetes		Diabetes^d^	
	UOR	95% CI	AOR	95% CI	AOR	95% CI	UOR	95% CI	AOR	95% CI	AOR	95% CI
**Wealth quintile**												
Lowest^b^												
Second	1.20	0.98–1.48	1.22	0.97–1.54	1.03	0.78–1.35	0.98	0.67–1.42	0.93	0.64–1.36	0.94	0.62–1.44
Middle	1.24§	1.00–1.54	1.20	0.96–1.51	1.09	0.83–1.42	1.01	0.72–1.42	0.94	0.67–1.32	0.80	0.52–1.22
Fourth	1.66*	1.36–2.02	1.54*	1.24–1.92	1.34§	1.03–1.75	1.46§	1.05–2.03	1.29	0.92–1.82	1.03	0.69–1.53
Highest	2.52*	2.07–3.07	2.18*	1.70–2.79	1.65†	1.22–2.25	2.83*	2.09–3.84	2.34*	1.65–3.32	1.81†	1.21–2.71
**Age group**												
35–39^b^												
40–44			1.49†	1.19–1.87	1.44	0.95–2.19			1.10	0.79–1.54	1.14	0.67–1.96
45–49			2.04*	1.63–2.55	1.83†	1.24–2.71			1.32	0.96–1.83	1.48	0.85–2.58
50–54			2.61*	2.05–3.31	2.83*	1.96–4.09			1.31	0.92–1.87	1.62§	1.01–2.60
55–59			2.73*	2.10–3.54	3.20*	2.18–4.69			2.29*	1.62–3.23	2.83*	1.72–4.66
60–69			4.00*	3.15–5.08	4.64*	3.21–6.69			1.44§	1.00–2.07	1.83§	1.13–2.97
70+			4.76*	3.69–6.15	5.94*	4.05–8.70			1.55§	1.05–2.30	1.98†	1.16–3.40
**Gender**												
Men^b^												
Women			2.23*	1.92–2.58	1.97*	1.59–2.46			1.09	0.88–1.35	0.90	0.66–1.25
**Currently married**												
No^b^												
Yes			0.80†	0.68–0.94	0.69†	0.56–0.85			0.92	0.70–1.22	0.86	0.60–1.23
**Schooling**												
None^b^												
Primary incomplete			0.88	0.74–1.03	0.87	0.71–1.07			1.28§	1.01–1.62	1.25	0.94–1.65
Primary complete			1.16	0.93–1.44	1.16	0.89–1.52			1.25	0.93–1.69	1.16	0.82–1.65
Secondary incomplete			1.38†	1.12–1.71	1.37§	1.03–1.82			1.48§	1.08–2.02	1.34	0.91–2.00
Secondary complete or higher			1.37§	1.03–1.84	1.73†	1.19–2.53			1.28	0.86–1.91	1.18	0.72–1.95
**Less physical labor**												
No^b^												
Yes			1.44§	1.07–1.94	1.13	0.82–1.56			1.40	0.96–2.03	1.35	0.89–2.06
**Place of residence**												
Rural^b^												
Urban			1.20§	1.03–1.40	1.14	0.95–1.38			1.00	0.78–1.28	0.97	0.73–1.28
**Body Mass Index (BMI)**												
Thin (BMI < 18.5)					0.53*	0.45–0.64					0.67†	0.50–0.89
Normal (BMI = 18.5–24.9)^b^												
Overweight (BMI = 25.0–29.9)					1.83*	1.46–2.89					1.74*	1.29–2.34
Obese (BMI ≥ 30.0)					3.14*	1.74–5.63					1.43	0.73–2.79
**L**	–4249.03		–3978.56		-2663.12		–2384.95		–2359.50		-1639.22	
**Δ–2X LL** ^**c**^	179.75*		540.94*		594.73*		171.72*		50.89*		83.18*	

Abbreviations: UOR, unadjusted odds ratio, AOR, adjusted odds ratio; CI, confidence interval; L, log-likelihood.

^a^ The first three models are for hypertension, and next three models are for diabetes. The log-likelihood ratio tests were performed with no sampling weight (without the Stata survey [svy] command).

^b^ Reference category.

^c^ Change in –2X log-likelihood when adding wealth to a model containing other variables. For the first model for hypertension and diabetes, respectively, it is the change when adding wealth to a model containing the constant only.

* P < 0.001;

^†^P < 0.01;

^§^ P < 0.05.

^d^ Total number of observations used are 5223.

**Fig 2:**
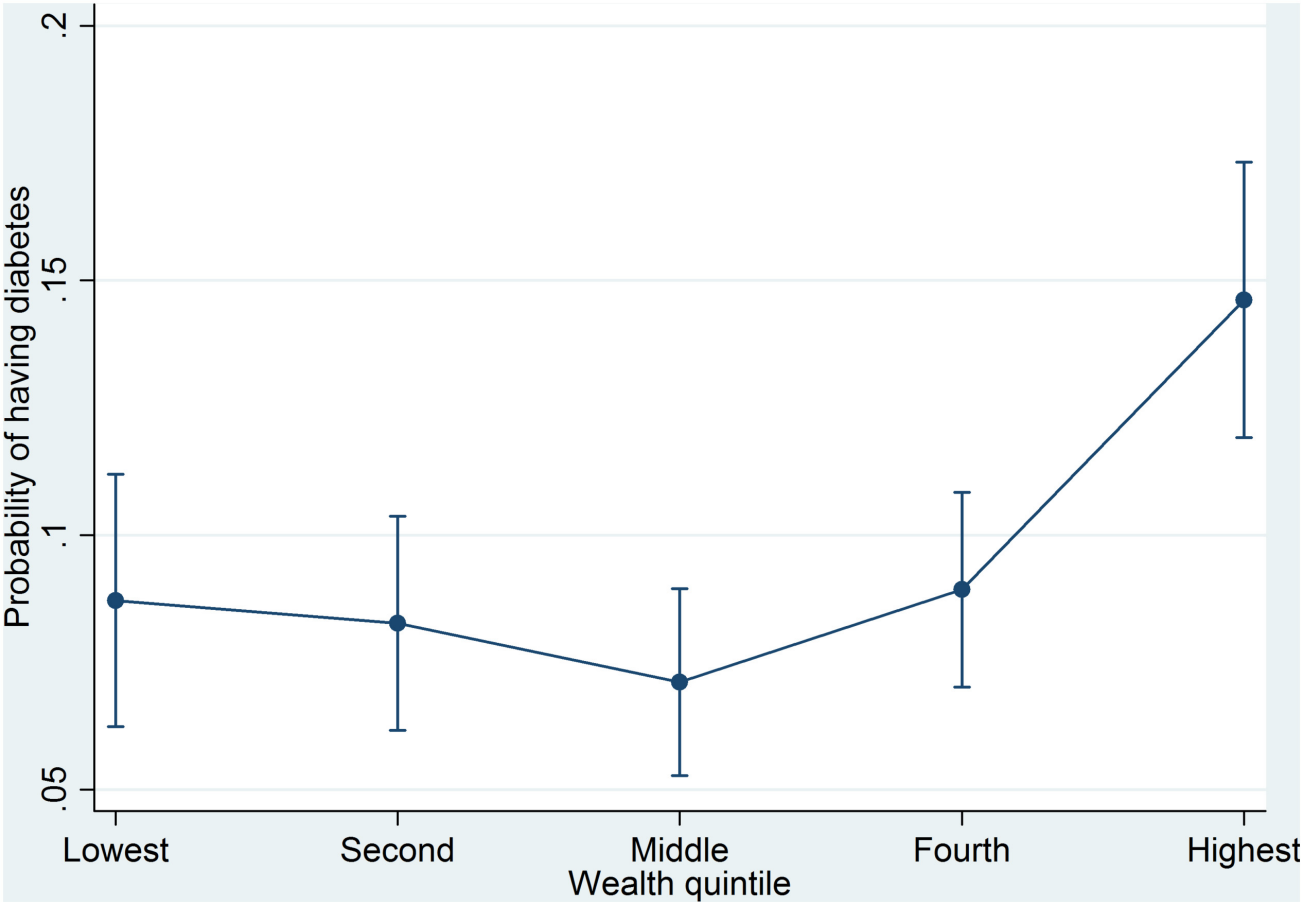
Predicted probabilities of having diabetes by wealth quintile.

**Fig 3:**
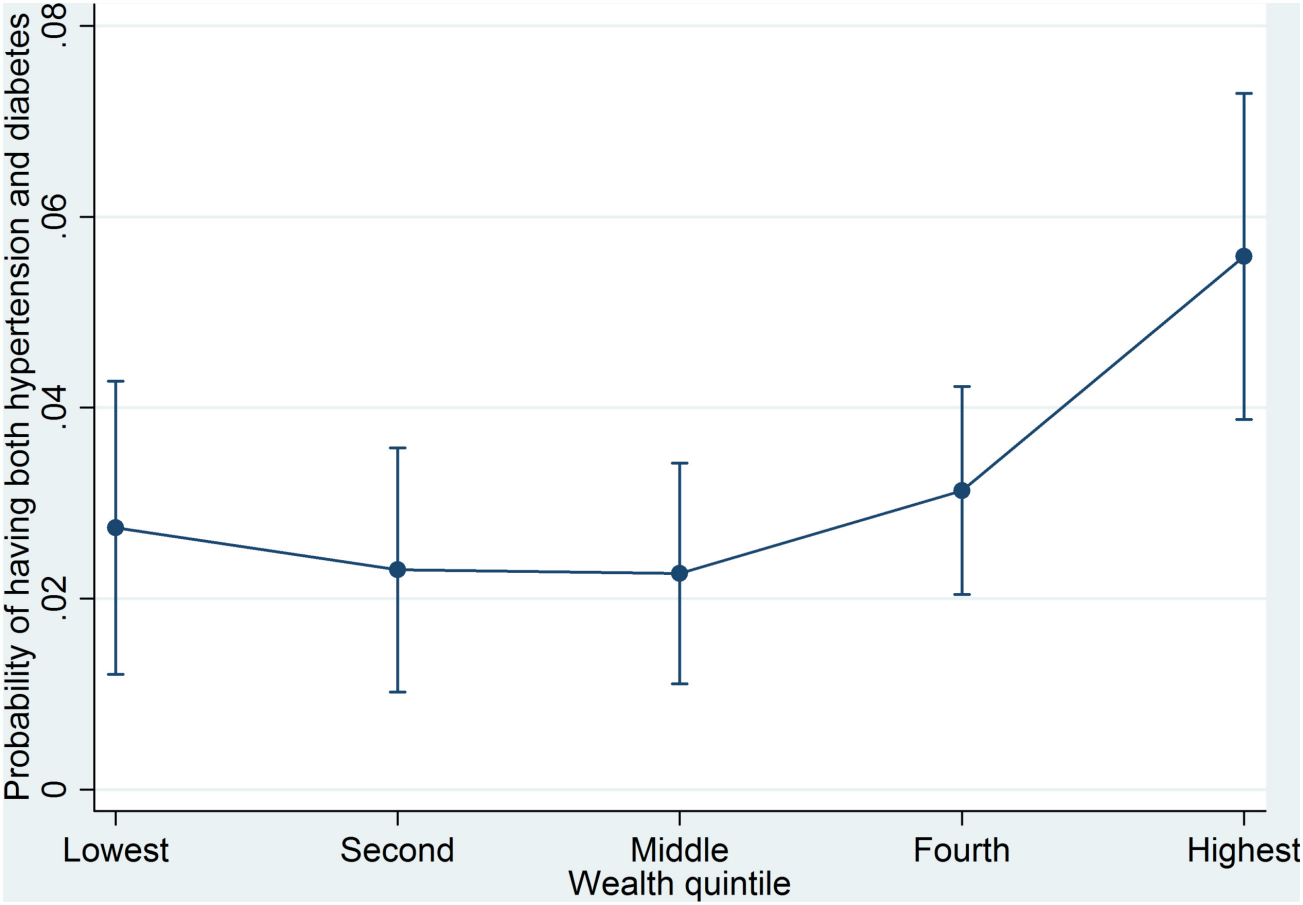
Predicted probabilities of having both hypertension and diabetes by wealth quintile.

Parallel results were obtained for diabetes and for the coexistence of hypertension and diabetes in Tables 3 and 4, which display an upward gradient for the association with wealth, except for the second and middle wealth quintiles; the probabilities of having diabetes and of the coexistence of hypertension and diabetes were higher for people from the fourth and highest wealth quintiles than for people from the lowest wealth quintile (Figs 2 and 3). In the fully adjusted model for diabetes (Table 3), people from the highest wealth quintile were significantly, 1.81 times (95% CI = 1.21–2.71), more likely to have hypertension than people from the lowest wealth quintile. In the fully adjusted model for the coexistence of hypertension and diabetes (Table 4), people from the highest wealth quintile were significantly, 2.17 times (95% CI = 1.05–4.49), more likely to have both hypertension and diabetes than people from the lowest wealth quintile.

**Table 4:** Odds ratios of having versus not having morbidity in Bangladesh (N = 7499)^a^.

	Both hypertension and diabetes		Both hypertension and diabetes		Both hypertension and diabetes^d^	
	UOR	95% CI	AOR	95% CI	AOR	95% CI
**Wealth quintile**						
Lowest^b^						
Second	1.16	0.60–2.27	1.09	0.56–2.12	0.83	0.38–1.82
Middle	1.28	0.67–2.47	1.21	0.58–2.15	0.82	0.38–1.77
Fourth	2.41†	1.35–4.28	1.86§	1.03–3.36	1.15	0.59–2.27
Highest	5.79*	3.38–9.92	3.68*	1.99–6.79	2.17§	1.05–4.49
**Age group**						
35–39^b^						
40–44			1.16	0.60–2.22	0.83	0.26–2.65
45–49			2.48†	1.43–4.31	1.68	0.67–4.19
50–54			2.55†	1.35–4.81	2.74§	1.16–6.43
55–59			5.76*	3.35–9.93	6.95*	3.14–15.39
60–69			4.57*	2.44–8.56	5.39*	2.37–12.27
70+			4.34*	2.30–8.18	5.84*	2.56–13.33
**Gender**						
Men^b^						
Women			2.16*	1.50–3.12	1.67	0.97–2.89
**Currently married**						
No^b^						
Yes			0.76	0.51–1.14	0.59§	0.36–0.99
**Schooling**						
None^b^						
Primary incomplete			1.35	0.89–2.03	1.15	0.71–1.87
Primary complete			1.43	0.86–2.37	1.48	0.84–2.60
Secondary incomplete			2.76*	1.62–4.69	2.27§	1.14–4.52
Secondary complete or higher			2.79†	1.38–5.63	3.26†	1.27–8.40
**Less physical labor**						
No^b^						
Yes			1.54	0.84–2.84	1.35	0.66–2.78
**Place of residence**						
Rural^b^						
Urban			1.12	0.78–1.62	1.06	0.69–1.62
**Body Mass Index (BMI)**						
Thin (BMI < 18.5)					0.25*	0.13–0.49
Normal (BMI = 18.5–24.9)^b^						
Overweight (BMI = 25.0–29.9)					1.92†	1.25–2.96
Obese (BMI ≥ 30.0)					1.55	0.69–3.50
**L**	–1111.33		–1060.22		-717.59	
**Δ–2X LL** ^**c**^	141.83*		102.23*		151.28*	

Abbreviations: UOR, unadjusted odds ratio, AOR, adjusted odds ratio; CI, confidence interval; L, log-likelihood.

^a^ The log-likelihood ratio tests were performed with no sampling weight (without the Stata survey [svy] command).

^b^ Reference category.

^c^ Change in –2X log-likelihood when adding wealth to a model containing other variables. For the first model, it is the change when adding wealth to a model containing the constant only.

* P < 0.001;

^†^P < 0.01;

^§^ P < 0.05.

^d^ Total number of observations used are 5223.

It can also be seen from Tables 3 and 4 that the disadvantages of being from a wealthier family for having hypertension and diabetes were attenuated, but persisted after incorporating controls for several covariates in the second and third set of models for all three outcomes. The odds of having all three outcomes increased at older ages. Women had greater odds than men of having hypertension and the coexistence of hypertension and diabetes. Currently married subjects had lower odds of having all three outcomes than those who were not currently married. People with some schooling were more likely to have all three outcomes compared to people with no schooling, with the exception of primary incomplete groups for hypertension. People doing less physical labor had higher odds of having the three outcomes than their counterparts. Compared to rural people, urban people were more likely to have hypertension and both hypertension and diabetes. People who were overweight and obese were more likely to have all three outcomes compared to people with normal BMI.

## Discussion

Only a handful of studies examine the relationship between economic wellbeing and hypertension and diabetes around the world. No previous study has tested an association between hypertension, diabetes, and the coexistence of hypertension and diabetes and economic wellbeing in Bangladesh. Given this gap, the current study reveals some crucial findings for global studies on hypertension and diabetes; namely the existence of a reversed gradient in having hypertension, diabetes, and the coexistence of hypertension and diabetes. People from the highest wealth quintiles demonstrated a greater likelihood of having hypertension, diabetes, and the coexistence of hypertension and diabetes than people from the lowest wealth quintiles. In Bangladesh, compared with the poor, the wealthy eat more, are over-nourished and more likely to be obese, do less physical labor and consequently develop hypertension and diabetes. Table 5 provides a possible reason why Bangladeshi people from the fourth and the highest wealth quintiles have greater odds of having all three outcomes: significantly higher percentages of respondents from the fourth and highest wealth quintiles were found to do less physical labor, and were more likely to be overweight and obese than their counterparts. Because early identification and preventive behavior for high blood pressure and elevated plasma lipid and blood glucose levels can reduce the risk of developing coronary heart disease, stroke and diabetes [27], the current study recommends that people from wealthier families, particularly from the fourth and highest wealth quintiles, in Bangladesh take proper measures to avoid developing hypertension and diabetes.

**Table 5:** Wealth distribution by socio-demographic characteristics.

	Wealth quintile				
Characteristics	Lowest	Second	Middle	Fourth	Highest
	%	%	%	%	%
**Gender**					
Men	19.92	19.37	19.37	20.52	20.81
Women	19.15	18.80	20.17	20.96	20.92
p value	0.539				
**Schooling**					
None	30.43	23.63	20.25	16.18	9.52
Primary incomplete	16.61	20.73	24.10	23.03	15.53
Primary complete	7.37	14.91	20.17	28.41	29.14
Secondary incomplete	4.58	11.97	15.65	25.78	42.02
Secondary complete or higher	0.58	3.29	10.35	24.40	61.37
p value	<0.001				
**Less physical labor**					
No	20.37	19.82	20.32	20.38	19.11
Yes	2.85	4.43	8.83	28.03	55.87
p value	<0.001				
**BMI** ^**a**^					
Thin (BMI < 18.5)	17.64	18.74	19.8	22.74	21.08
Normal (BMI = 18.5–24.9)	29.52	24.73	21.76	15.66	8.33
Overweight (BMI = 25.0–29.9)	3.62	7.04	14.39	24.68	50.27
Obese (BMI ≥ 30.0)	2.47	6.48	4.28	27.81	58.96
p value	<0.001				

p values are from Chi-square tests.

^a^ Total number of observations used are 5223.

Assuming that the association runs from wealth to having hypertension and diabetes, it is quite possible that a series of socio-demographic factors intervene. A positive association between age and hypertension and diabetes is reported in several studies [16, 18, 28, 29]; the prevalence of hypertension and diabetes increases as age increases. Our study also shows that older people have a higher prevalence and odds of having hypertension, diabetes, and the coexistence of hypertension and diabetes than younger people.

The possibility of women developing high blood pressure increases as they grow older and becomes higher than that of men. The greater probability of having hypertension in women results from oral contraceptive use [30, 31] and menopause [31]. Compared with men, women had a significantly greater likelihood of hypertension and the coexistence of hypertension and diabetes in the current study, which should encourage women to be careful to avoid developing those risk factors that are precursors of disease.

Marriage is protective of health in general and this is especially true among older couples [32, 33]. In the present study, currently married people were less likely to have hypertension and/or diabetes. People with lower education and/or from lower occupational categories are reported to have a higher prevalence of hypertension [2] and diabetes [9]. However, a recent meta-analysis reported an inverse association between education and hypertension in rural East Asia, and a positive association in rural South Asia [15]. Our study found that people with some schooling have a significantly higher prevalence of hypertension, diabetes and the coexistence of hypertension and diabetes than people with no schooling. In addition, people who engage in less physical labor have a higher likelihood of having hypertension and the coexistence of hypertension and diabetes than those who do not. Therefore, this study also recommends taking initiatives against developing hypertension and/or diabetes for those who have some education and who work in professional occupations that do not require physical labor, such as doctor lawyer, dentist, accountant, teacher, nurse, family welfare visitor, big businessperson, and imam or religious leader.

The risk of hypertension was reported to be higher among populations who are overweight and obese in Ethiopia, Vietnam and Indonesia [34]. Increased BMI was also reported to be associated with increased prevalence of hypertension and diabetes in the US [35, 36]. A study with limited sample size in Dhaka, Bangladesh also reported a positive association between diabetes, being overweight and obese [18]. The current study also reveals that people who were overweight and obese were more likely to have hypertension, diabetes and the coexistence of hypertension and diabetes than people with normal BMI. Therefore, this study also recommends Bangladeshi people to adopt a healthier life style, avoid a sedentary life style and work to keep their BMI within a normal range to prevent developing hypertension and/or diabetes.

Although a higher percentage of men than women have some schooling and do less physical labor (Table 1), men have lower odds ratios of having hypertension and the coexistence of hypertension and diabetes than women (Tables 3 and 4). It might be due to a comparatively higher percentage of women in the middle to highest wealth quintiles than that of men (Table 5) and a comparatively higher percentage of women who were overweight and obese than that of men (Table 1).

## Strengths and weaknesses

This study has two major strengths. First, to our knowledge, it is the first study that examines inequality in the prevalence of hypertension, diabetes, and their coexistence in Bangladesh using a nationally representative dataset. Second, the most recent dataset used in this study provides us with objective measures of hypertension, diabetes and BMI. However, the current study was constrained by the preexisting variables in the 2011 BDHS. We could not explore the effect of exercise behavior, or stress on the three outcomes due to lack of data. Nurses and family welfare visitors should not be in the category of less physical labor, because many nurses and family welfare visitors are quite physically active in Bangladesh. But we were constrained by the built-in codes of the 2011 BDHS dataset. In the 2011 panel, doctor, lawyer, dentist, accountant, teacher, nurse and family welfare visitor were kept as a single code. The study also does not provide any information about people under the age of 35 because the 2011 BDHS only collected measurements on hypertension or diabetes for people aged 35 years and above.

## Conclusions

Unlike the existing literature on inequalities in health throughout the world, the current study shows that the prevalence of hypertension, diabetes, and the coexistence of hypertension and diabetes are higher in the upper wealth quintiles in Bangladesh. Therefore, in Bangladesh, this study recommends that wealthier people be careful to avoid unhealthy habits and life styles. In respect to hypertension and diabetes, health policy makers and planners are urged to prioritize programs that target the kinds of habits made possible by wealth while also paying special attention to older people, women, people who engage in less physical labor, and individuals who are overweight and obese.
